# Chemical probing of HER2-amplified cancer cells identifies TORC2 as a particularly effective secondary target for combination with lapatinib

**DOI:** 10.18632/oncotarget.5660

**Published:** 2015-10-14

**Authors:** Dhara N. Amin, Ana Ruiz-Saenz, Nathaniel Gulizia, Mark M. Moasser

**Affiliations:** ^1^ Helen Diller Family Comprehensive Cancer Center, University of California, San Francisco, CA 94143, USA

**Keywords:** HER2, HER3, TORC2, mTOR, lapatinib

## Abstract

The clinical impact of HER2 inhibitors in the treatment of HER2-amplified breast cancers has been largely confined to chemotherapy combination regimens, since HER2 inhibitors appear to have very modest efficacies by themselves. This is due to the resilient nature of the functionally relevant HER2-HER3 tumor driver, bidirectionally linked with downstream PI3K/Akt pathway signaling, which can break through the inhibitory effects of most current HER2 or HER3 targeting therapies. A vertical combination approach targeting HER2 and a downstream pathway is a highly rational strategy for much more effective targeted therapy of this disease. However the importance of these downstream pathways in many human tissues and cells significant limits their usefulness as secondary targets by narrowing the therapeutic index of such combination therapies. The secondary target that can afford the highest potential for clinical translation is the one with the highest synergy against tumor cells in combination with HER2-inhibition, allowing the widest therapeutic index for clinical translation. We conducted a comparative analysis of such secondary targets in combination with the HER2 inhibitor lapatinib and find that the inhibition of mTor affords the highest degree of synergy. In further dissecting the individual roles of TORC1 and TORC2 complexes using pharmacologic and genetic tools, we find that it is specifically the inactivation of TORC2 that most synergistically enhances the efficacy of lapatinib. Although inhibitors that selectively target TORC2 are not currently available, these data make a compelling case for their development.

## INTRODUCTION

The treatment of oncogene-driven cancers through the direct inactivation of their driving oncogenes is a treatment strategy rooted in solid scientific rationale. This treatment strategy has now become a mainstay of cancer therapeutics, at least in the realm of kinase oncogenes, since pharmaceutical technologies are now readily available to inhibit kinases with relative selectivity and potency. Such kinase inhibitors have revolutionized the treatment of lung cancers driven by mutationally activated EGFR, Alk, or Ros, or melanomas driven by mutationally activated BRAF, or leukemias driven by mutationally activated Abl [1–5]. However the treatment of HER2-amplified cancers has not followed the same paradigm. Although the clinical kinase inhibitors developed for this disease represent some of the best in class with respect to selectivity and/or potency, their clinical anti-cancer activities are modest at best [6–11]. This is due to the inherent complexity in the HER2 target that sets it apart from many of the other kinase oncogenes.

HER2, by its very nature, signals in a codependent manner and its partner HER3 plays a key role in mediating many of its biological functions. In fact, the function of HER3 is essential in HER2-driven tumorigenesis, now confirmed in several models of HER3 knockdown in tumor cells and conditional HER3 knockout in mouse models [12–14]. Although HER3 is kinase inactive, it is an important mediator of HER2 signaling, particular because of its ability to activate the downstream PI3K/Akt pathway [15, 16]. But the dependency on HER3 is not just a passive dependency as an important second messenger. Rather, it's a bidirectional codependency such that inhibiting HER2 actually induces a robust increase in HER3 expression and signaling, through a multitude of mechanisms, that are able to amplify HER2-HER3 signaling [17–19]. The dynamic range of signaling by the HER2-HER3 tumor-driver is about two logs, thus a near complete inhibition of the HER2 kinase is required for effective tumor cell killing [20]. While this can easily be achieved *in vitro* by fully inactivating concentrations of HER2 inhibitors, it remains beyond the therapeutic index of all such agents in the clinical setting. Combination therapy approaches afford a promising direction for further pursuit.

Although HER3 itself is an ideal secondary target for the treatment of HER2-amplified cancers, it is currently not an easily druggable target and it may be years before the structural basis of its functions can be understood and potently inhibited by appropriately designed drugs. However, the signaling cascade downstream of HER3 involves a number of kinases including PI3K, Akt, and mTor, which are the targets of a plethora of kinase inhibitors in the pharmaceutical pipelines and in early-mid phases of clinical study. But these kinases play fundamentally important roles in many cellular functions and downstream of many tyrosine kinase receptor families, and these targets may not afford high therapeutic indices for targeting, except perhaps in cancers wherein they specifically function as the oncogenic driver due to genomic alterations. We have explored the potential of downstream kinases as secondary targets for combination with HER2 inhibitors in the treatment of HER2-overexpressing cancers. Although all combination therapies often afford additive benefits in cell-based assays, it is the combinations with the highest synergies that are deemed most likely to provide a wide enough therapeutic index to substantially improve clinical efficacy. Our analysis here highlights the potential of mTor, and in particular the mTor complex-2 (TORC2), which appears to be the most promising target for combination therapy approaches.

## RESULTS

**Figure 1:**
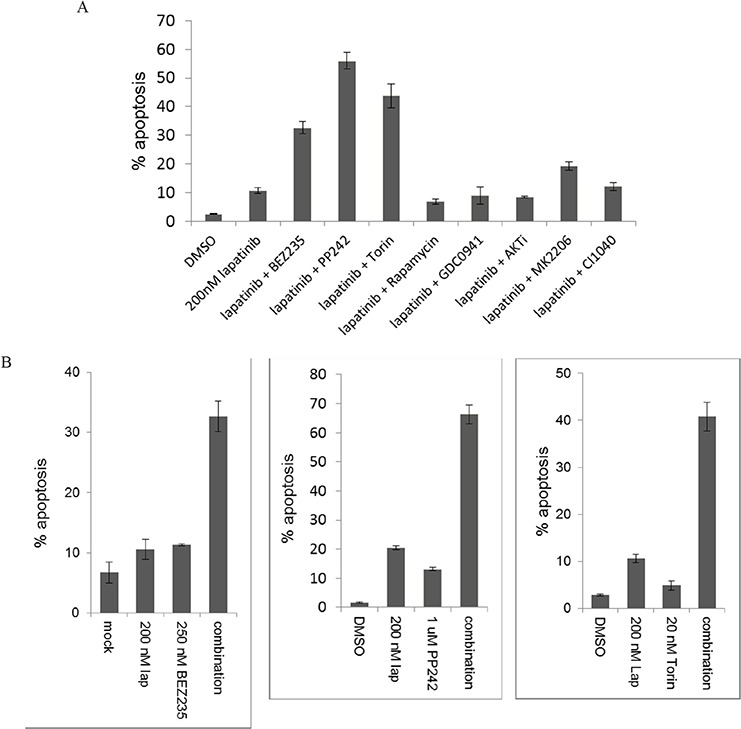
The apoptotic efficacy of lapatinib in combination with downstream targeting **A.** SKBr3 cells were treated with the HER2 inhibitor lapatinib (200 nM) by itself or in combination with inhibitors of PI3K (0.5 uM GDC0941), dual PI3K/mTor (250 nM BEZ235), mTor kinase (1 uM PP242 or 20 nM Torin1), TORC1 (25 nM rapamycin), AKT (1 uM AKTi or 2.5 uM MK2206), or MEK (100 nM CI1040) for 72 hours. The fraction of apoptotic cells was assayed by FACS analysis of DNA degradation. Results are average of triplicates and error bars represent S.E.M. **B.** For the inhibitors of mTor kinase, the experiments were repeated using single and combination therapy arms for 72 hours.

We have previously shown that treatment of SkBr3 cells with 200 nM lapatinib induces growth arrest, but fails to induce apoptotic cell death due to the failure to durably suppress downstream signaling [17, 20]. This is primarily a failure to inhibit signaling along the HER3-PI3K-Akt-mTOR pathway, and we have previously shown that it is due to robust compensatory negative feedback signaling that functions to protect and preserve the continuity of this signaling pathway, well known to be critical for many aspects of tumorigenic growth [18, 20]. A rationale idea for more effective therapy would be the use of a vertical combination therapy approach that targets two points along this pathway, encompassing HER2 as well as one of the downstream signaling nodes. We tested this concept by screening a number of drugs targeting these downstream kinases for their ability to induce apoptosis when added to 200 nM lapatinib. This concentration of lapatinib was chosen for this screen because it transiently inhibits HER2-HER3 signaling and induces growth arrest, but is overpowered by the compensatory mechanisms driven by downstream HER3/PI3K/Akt signaling and fails to induce tumor apoptosis [20]. The second drugs were chosen from among many available tool compounds and clinical agents targeting PI3K, Akt, and mTOR. The sites of activity of these drugs and references to their biochemical characteristics are provided in Supplementary figure 1. Two drugs were tested for each target for reproducibility, and at least one clinical compound used for each target to enable clinical translation of any promising results. The concentrations of each second drug was selected from pilot experiments that were designed to identify low concentrations that completely inactivated their direct targets in these cells. Of these lapatinib combinations, the BEZ235, PP242, and Torin1 combinations showed significantly more apoptosis than lapatinib alone (figure 1A). The apoptosis seen with these lapatinib combinations is not simply from the activity of the second drug alone, as determined by separate experiments showing the pro-apoptotic activities of each drug compared with their combinations (figure 1B).

**Figure 2:**
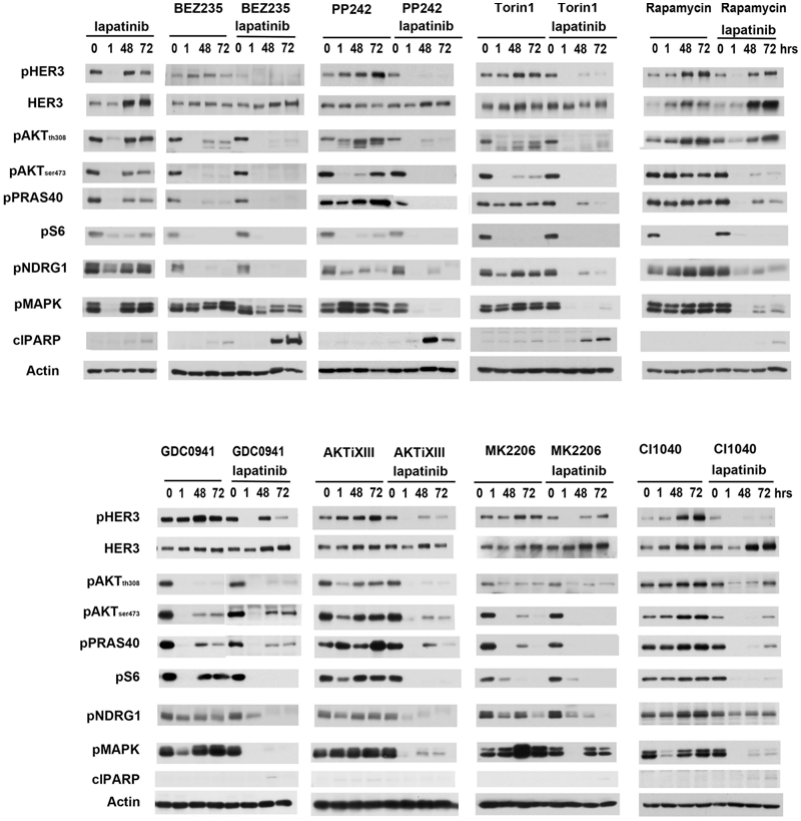
The effects of lapatinib combinations on downstream signaling SKBr3 cells were treated with either 200 nM lapatinib alone or in combination with PI3K, AKT, mTor and MEK inhibitors. Cell lysates were collected at 0, 1, 48, 72 hours post-treatment and analyzed by western blotting for phosphorylation of proteins at various signaling nodes downstream of HER2 signaling. Drug concentrations are identical to figure 1.

We also assayed the effects of these drug combinations on downstream signaling (figure 2). Lapatinib alone effectively inhibits HER3 phosphorylation and downstream MAPK signaling and PI3K/Akt/mTor signaling, when examined at the 1 hour timepoint. However, this inhibition is not durable and the new steady state seen at 48 hours of continued therapy exhibits increased HER3 expression and restored signaling throughput. Of the combinations studied, the combinations with BEZ235, PP242, and Torin1 afford the most effective suppression of HER3 signaling. Consistent with more effective suppression of oncogenic signaling, PARP cleavage is also seen with these combinations, indicating activation of the apoptotic signaling cascade.

**Figure 3:**
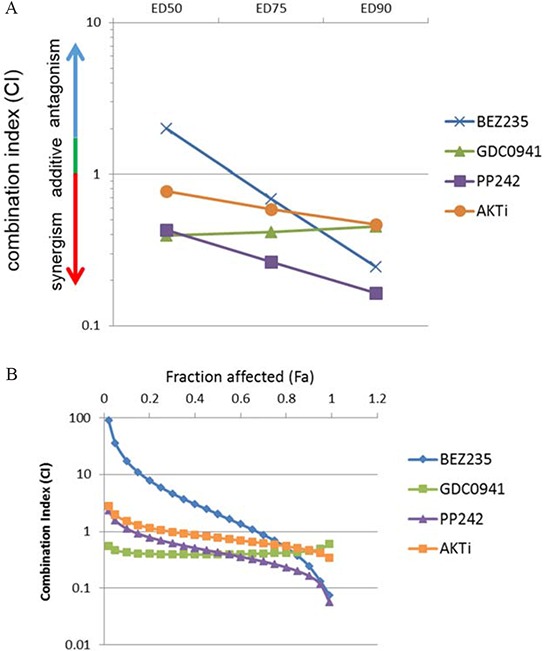
mTOR kinase inhibitors show the highest extent of synergy in combination with lapatinib **A.** SkBr3 cells were treated with increasing concentrations of lapatinib and the indicated second drugs at an equimolar drug ratio in a matrix format for 72 hours and the combination indices were calculated. The CI is plotted against effective dose values that resulted in 50%, 75% and 90% effects on viability. **B.** The CI is plotted against a continuous range of fraction affected (Fa) values. CI < 0.9 synergy, CI > 1.1 = antagonism and CI 1 = additivity.

We performed additional experiments to better characterize and quantify how each drug combined with lapatinib. Drug combination studies with the inhibitors were performed in a matrix format and IC50 values of the drugs for inhibiting SKBr3 cells are provided (Supplementary Table 1). Dose response curves show that for equimolar drug ratios the combinations of the inhibitors with lapatinib was superior to inhibition with single drug (Supplementary figure 2). For quantifying synergy between two drugs we performed Combination Index (CI) analysis of the drugs in combination with lapatinib according to the method of Chou & Talalay wherein synergy is defined by a CI value of less than 0.7; with decreasing CI values indicating greater synergy [21, 22]. The combination index was obtained at three different equimolar concentrations and the CI plots showed that the mTOR kinase inhibitor PP242 displayed the highest synergy for inhibiting growth in combination with lapatinib (figure 3A).

We also calculated the CI values across the entire range of concentrations generating the CI plot against the Fraction Affected (Fa) spectrum. Fa-CI plots also show that the mTOR inhibitors display the highest synergy at high Fa values (Figure 3B). For cancer drugs, CI values at higher Fa values are more appropriate than the lower Fa [21]. This analysis was performed using fixed ratios of drugs at equimolar concentrations. However, the use of different drug ratios also supports that PP242 shows the highest synergy in combination with lapatinib compared to PI3K or AKT inhibitors (Supplementary figure 3).

**Figure 4:**
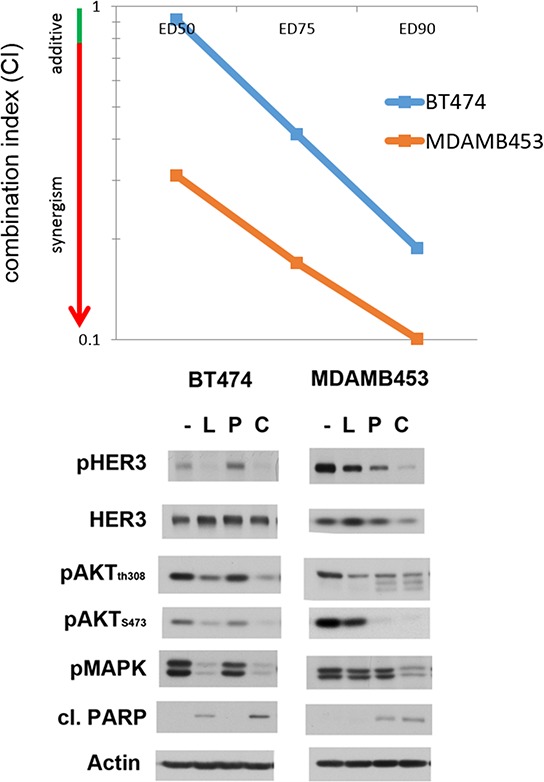
Lapatinib and mTor inhibitor combination in other HER2-amplified cell lines **A.** BT474 and MDA MB 453 cell lines were treated with lapatinib in combination with the mTOR inhibitor PP242 for 72 hours at increasing concentrations of the drugs at an equimolar drug ratio. Combination Index value is reported for the two lines at effective dose (E.D.) values of 50, 75 and 90%. **B.** BT474 and MDA MB453 cells were treated with DMSO (−), 200 nM lapatinib (L), 1 uM PP242 (P) or the combination (C) for 72 hours. Cell lysates were analyzed by western blotting for phosphorylation of signaling proteins downstream of HER2.

To determine whether the synergistic effects of lapatinib and PP242 are evident in other HER2-amplified cancer cell lines, we tested them in equimolar ratios in 3 more cell lines. All show increased efficacy with the combination (Supplementary figure 4). We specifically determined the CI of the lapatinib and PP242 combination in BT474 and MDA-MB-453 cells at three concentrations, confirming the synergistic interaction of this combination in these cell lines as well (figure 4A). We also studied the effects of the lapatinib/PP242 combination on downstream signaling in BT474 and MDA-MB-453 cells. In both cells, the combination showed greater suppression of downstream signaling compared with individual drugs (figure 4B).

**Figure 5:**
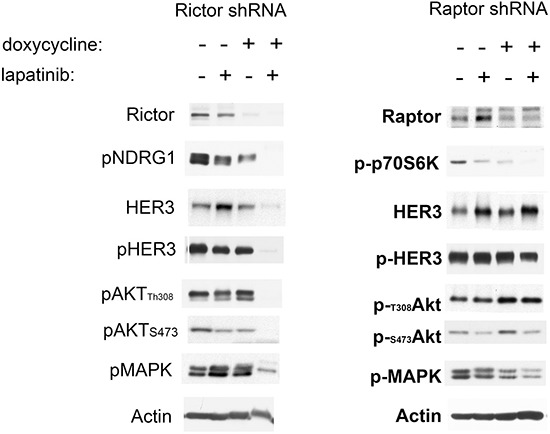
Lapatinib in combination with TORC1 or TORC2 knockdown **A.** SKBr3 cells expressing dox-inducible shRNAs targeting either Rictor or Raptor were treated with or without doxycycline (6 days for Rictor and 3 days for Raptor) and in the presence or absence of 200 nM lapatinib for 72 hours Cell lysates from the treated cells were analyzed by western blotting as indicated. P-p70S6K is a readout of TORC1 signaling, whereas P-NDRG1 is a readout of TORC2 signaling.

The data above shows that the efficacy of lapatinib against HER2-amplified cancer cells can best be enhanced by the addition of either BEZ235, PP242, or Torin1. What these drugs all have in common is that they are of the ATP-analog class of mTor kinase inhibitors. BEZ235 also inhibits PI3K, but what is common between them is the inhibition of mTor kinase activity. This kind of synergy is not seen with the allosteric mTor inhibitor rapamycin (figure 1A). Since rapamycin has only limited effects on the functions of mTor, principally targeting some mTor function in its TORC1 complex [23, 24], we decided to more specifically query the roles of TORC1 and TORC2 as synergistic targets in combination with HER2. For these experiments we established SkBr3 cells with tet-inducible shRNA knockdown of either Raptor or Rictor to target either TORC1 or TORC2 functions. The time courses of knock down of Raptor and Rictor following doxcycyline treatment were established in initial pilot experiments (Supplementary figure 5), and for combination experiments lapatinib treatment was instituted at the time of effective Raptor or Rictor protein suppression. The concomitant knockdown of Raptor with lapatinib showed no significant suppression of downstream signaling (figure 5). However, the concomitant knockdown of Rictor significantly enhanced the effects of lapatinib on downstream signaling (figure 5). The inactivation of TORC2 by Rictor knockdown prevents the compensatory induction of HER3 expression seen with lapatinib therapy and consequently leads to a much more complete inactivation of Akt by lapatinib. Similar results were seen when TORC2 was targeted in BT474 cells and in MDA-MB-453 cells (Supplementary figure 6).

**Figure 6:**
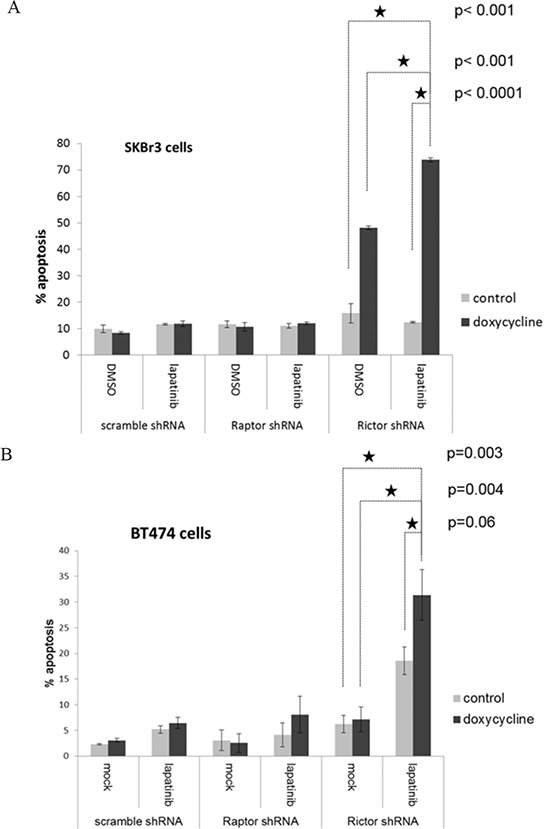
The apoptotic efficacy of lapatinib in combination with TORC1 or TORC2 targeting **A.** SKBr3 cells stably expressing dox-inducible inactivation of TORC1 (by Raptor shRNA) or TORC2 (Rictor shRNA) were treated with or without 200 ng/ml doxycycline, and upon knockdown of Raptor or Rictor, were also treated for an additional 72 hours with or without lapatinib. The timepoints of Rictor and Raptor knockdown were previously determined in pilot experiments. After 72 hours of lapatinib treatment, apoptotic fraction was assayed by FACS analysis of DNA degradation. The data shown is the mean of triplicates and error bars represent S.E.M. The P values for the comparisons indicated by the stars is shown to the right of the stars. **B.** The identical experiment is shown for BT474 cells.

To determine whether co-targeting TORC2 significantly enhances the apoptotic effects of lapatinib, we assayed the effects of dual HER2/TORC2 targeting on cell death. Interestingly, targeting TORC2 by itself induces significant apoptotic cell death in SkBr3 cells (figure 6A). however the addition of lapatinib significantly enhances the apoptotic effects of targeting TORC2. In BT474 cells, disruption of TORC2 function by itself does not induce apoptosis, but it does significantly enhance sensitivity to lapatinib-induced apoptosis (figure 6B).

## DISCUSSION

The treatment of HER2-amplified cancers has evolved considerably over the past two decades as we have developed a much better understanding of the functions of HER2, the structural basis for its functions, and the signaling context within which it function. The HER2-targeting biological agents developed and introduced into clinical practice have made considerable impact and additional agents, currently in the investigational pipelines, will likely enter the clinical realm in the near future. However the clinical activities of HER2-targeting agents without chemotherapy components remain in the incremental range, and the highly effective treatment of HER2-driven cancers through inhibition of HER2 signaling has proven more challenging than other oncogene-driven cancers. This is because of the resilient signaling network within which it functions and its highly robust ability to compensate for anything short of the complete inhibition of HER2 signaling [20]. At this point it seems like the most promising approach to effectively disrupt this oncogenic signaling pathway is by dual targeting. The concept of dual targeting is simple in design, but inherently difficult in translation and implementation. This is because of the inherent toxicities frequently associated with broader targeting and the resulting reduced therapeutic index associated with broader targeting that significantly limits the clinical efficacies of such combination therapy approaches. While the massive overexpression and overactivity of HER2 is highly unique to these cancer cells, the activities of many of the downstream signaling elements linked with it such as PI3K, Akt, MAPK, and mTor are less unique to cancer cells. Their function is physiologically engaged by upstream activation of HER2, not pathologically engaged through mutation or amplification, and although their activity levels are increased as a consequence of HER2 overactivity, this represents only a relative increase in activity compared to many cells and tissues in the body that also depend on these signaling proteins for their functions. As such, their use as secondary targets can considerably reduce the therapeutic index of such combination therapies. However such secondary targets may have differential levels of importance in the circuitry engaged by HER2 amplification and the best secondary target would be the one with the most critical role in the cancer cells, a so called “Achilles heel” type of secondary target. Such a target, in theory, could afford the widest therapeutic index and have the highest likelihood of success in combination with HER2 targeting. In this work, we screened a number of secondary targets using available kinase inhibitors and we find that the target that affords the highest combinatorial index with a HER2 inhibitor is mTor.

The concept of therapeutic index is critical to clinical translation. The clinical efficacy of any drug or drug combination is not only related to its direct effects on cancer cells, but also its effects on other host tissues. This is because the drugs effects on other tissues creates toxicities that limit the dose that can be administered and ultimately limits the target inactivation within cancer cells. Combinations of drugs are likely to have a narrower therapeutic index because of the wider range of targets inhibited in the normal host tissues and the increase in toxicities that limits their dosing. However drug combinations that show synergy against cancer cells are particularly attractive candidates for clinical translation. This is because synergy is not a global phenomenon, and is a consequence of the existing circuitry and network encompassing the two targets. Since the signaling circuitry in cancer cells is frequently altered, synergism in cancer cells may be specific to cancer cells and not relevant to the host tissues, thereby providing a widening of the therapeutic index.

The importance of the PI3K/Akt/mTor signaling pathway in HER2-amplified breast cancers has been known for some time, and the idea of combining inhibitors of HER2 with inhibitors of this downstream pathway has been studied and showed superior activity in a number of preclinical studies [25–28]. Not all these combinations have been tested and reported in clinical studies yet, but combinations with the TORC1 inhibitor everolimus show only modest activity [29, 30]. We undertook a broader comparative and quantitative analysis of several different vertical combination stategies which we carried further by dissecting the differences between TORC1 and TORC2 as secondary targets.

mTor has critical functions in cellular homeostasis and is important in many cells and tissues, and its addition to HER2 inhibitors is likely to significantly diminish the therapeutic index of the combination. Indeed in early phase clinical studies mTor kinase inhibitors have shown little signals of efficacy, and their dosing has been limited by organ toxicities [31, 32]. However mTor itself is a multifunctional target and thus it is possible to target its functions selectively. mTor exists in two large multi-protein complexes which appear to have different functions [33]. The functions of TORC1 are better known and include the regulation of protein synthesis and translation, whereas the functions of TORC2 are currently less well understood. The activities of mTor are regulated by the proteins it interacts with which are different in its two complexes. Drugs that inhibit the catalytic kinase activity of mTor, such as PP242 and Torin1 and other similar drugs in the pharmaceutical pipelines, inhibit all of its functions in both of the mTor complexes. However the fact that protein allostery regulates the signaling functions of the individual mTor complexes suggests that allosteric type inhibitors can be designed to inhibit the functions of the mTor complexes selectively. The prime example of this is rapamycin and the variety of its analogues that inhibit only the functions of TORC1 [23, 24]. Rapamycin binds the cytosolic protein FKBP12 and creates a ternary complex specifically with TORC1, inhibiting the function of mTor only in this complex [34]. It remains plausible that future work can identify allosteric classes of drugs that selectively interfere with the functions of TORC2. Our results provide a highly compelling case for the development of such inhibitors. The studies presented here using shRNA targeting of TORC2 suggest that HER2-amplified tumors are particularly sensitive to the loss of TORC2 function, with considerable apoptotic cell death ensuing with the loss of TORC2 alone (such as in SkBr3 cells) or with considerable synergy when combined with the HER2 inhibitor lapatinib (such as in BT474 cells).

## MATERIALS AND METHODS

All cell lines were purchased from American Type Culture Collection and cultured at 37C, 5% CO2 in DMEM/HamF12 media supplemented with 10% fetal bovine serum, penicillin, streptomycin, and L-glutamine. The AKT allosteric inhibitor AKTi (Akt XIII) was purchased from Sigma. BEZ235 was obtained from Novartis. Lapatinib was purchased from the pharmacy as tablets and the active ingredient purified by organic extraction as previously described [20]. CI1040 was from SYN thesis med chem, Rapamycin was from Cell Signaling, Torin1 and Mk2206 were from SellekChem. GDC0941 and PP242 were provided by Kevan Shokat. All pharmaceutical drugs were reconstituted in DMSO.

Growth assays were done in matrix format. Cells (2000/well) were plated in 96 well plates and were allowed to adhere overnight. The cells were treated with increasing doses (11 nM–2700 nM) for all drugs except BEZ235 and Rapamycin (3–900 nM) in a 3-fold increasing matrix format. After 72 hours MTT reagent (0.42 mg/ml) was added to the cells for 4 h hrs and the cells lysed in DMSO and absorbance read at 570 nm. The percentage absorbance upon treatment compared to DMSO treatment is reported as an average of triplicates. Error bars represent S.E.M. For combination index analysis, the data from the MTT assays was analyzed using the Calcusyn software. The drugs were at fixed ratios of 1:1, 1:3, or 3:1 for lapatinib:drug X. The Calcusyn software analyses for synergy, additivity or antagonism between two drug combinations based on the Chou & Talay methodology.

Apoptosis assays. To quantitate the amount of apoptotic cell death, FACS analysis of nuclear degradation was performed as described [20]. % Sub G1 cells are reported from three independent repeats of experiments. Error bars are calculated from S.E.M. Student's *t*-test were performed and *p*-values reported.

Cell lysates were prepared using modified RIPA buffer supplemented with protease and phosphatase inhibitors. Western blots were performed using antibodies purchased from SantaCruz Biotechnologies (HER3, actin), Cell Signaling (p-HER2, pAKTth308, pAKTser473, pS6, p-NDRG1, p-MAPK, Rictor, Raptor), Enzo Life Sciences (pPRAS40). The custom made anti-pY1289-HER3 was previously described [20].

For inducible knockdowns of Rictor and Raptor and Scramble-control knockdowns, cells were first transfected with the pcDNA6/TR to express the tetracycline repressor, and subsequently infected with the pSuperior vector carrying the inducible shRNA sequences. The below described oligonucleotides were synthesized and annealed and then ligated into pSuperior vector. Standard protocols were used according to the manufacturers (OligoEngine). shRNA sequences used are described below:

### Rictor shRNA sequences

5′-GATCCCGCAGCCTTGAACTGTTTAATTCA AGAGATTAAACAGTTCAAGGCTGCTTTTTGGAA A-3′

5′-AGCTTTTCCAAAAAGCAGCCTTGAACTG TTTAATCTCTTGAATTAAACAGTTCAAGGCTGCG G-3′

### Raptor shRNA sequences

5′-GATCCCAGGGCCCTGCTACTCGCTTTTCA AGAGAAAGCGAGTAGCAGGGCCCTTTTTTGGAA A-3′

5′-AGCTTTTCCAAAAAAGGGCCCTGCTACTC GCTTTCTCTTGAAAAGCGAGTAGCAGGGCCCTGG-3′

### Scramble shRNA sequences

5′-GATCCCCCTAAGGTTAAGTCGCCCTTTCA AGAGAAGGGCGACTTAACCTTAGGTTTTTGGAA A-3′

5′-AGCTTTTCCAAAAACCTAAGGTTAAGTCG CCCTTCTCTTGAAAGGGCGACTTAACCTTAGGGG-3′

The doxycycline-induced models were thoroughly investigated to determine the timeline of gene induction or suppression following doxycycline treatment in order to query the signaling throughput at timepoints before and at early and late timepoints after the genetically induced events.

## SUPPLEMENTARY FIGURES AND TABLES


