# Supplementary material for: Chemical probing of HER2-amplified cancer cells identifies TORC2 as a particularly effective secondary target for combination with lapatinib
Source: Oncotarget. 2015 Oct 14;6(38):41123–33. doi: 10.18632/oncotarget.5660 (PMC4747394; doi:10.18632/oncotarget.5660)
Supplement: Supplementary file 1 [file oncotarget-06-41123-s001.pdf]

SUPPLEMENTARY FIGURES AND TABLES

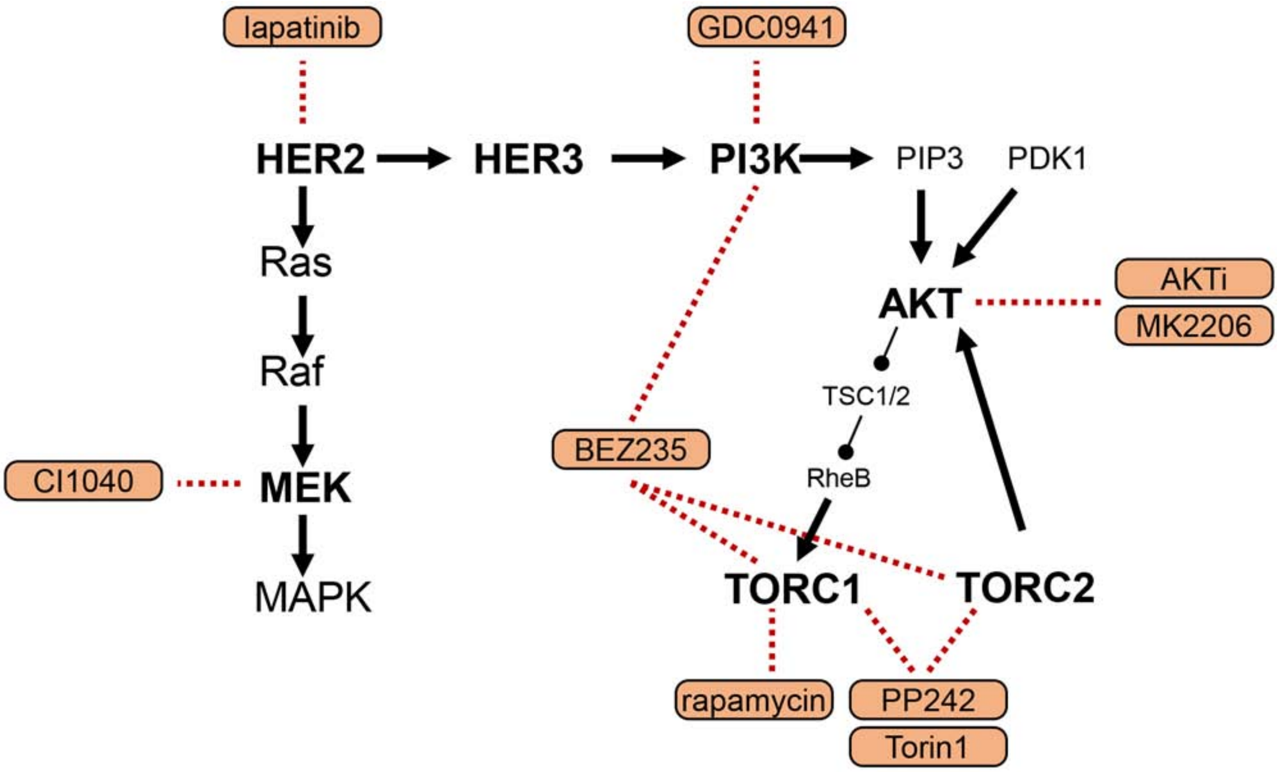

Drug	Reference
lapatinib	Rusnak et al, Mol Cancer Ther 2001; 1: 85-94.
GDC0941	Folkes et al, J Med Chem 2008; 51: 5522-32.
BEZ235	Maira et al, Mol Cancer Ther 2008; 7: 1851-63.
Akti	Zhao et al, Bioorg Med Chem Lett 2008; 18: 49-53.
MK2206	Yan. Cancer Res 2009; 69; abstract DDT01-1
rapamycin	reviewed in Faivre et al, Nat Rev Drug Discov 2006; 5: 671-88.
PP242	Feldman et al, PLoS Biol 2009; 7: e38
Torin1	Liu et al, J Med Chem 2010; 53: 7146-55.
CI1040	Bioorg Med Chem Lett 2008; 18: 6501-4.

Supplementary Figure S1: Schematic depicting the general outline of the signaling pathway downstream of HER2-HER3 and the kinases targeted by the individual drugs. Readers are referred to the following references for the biochemical properties and available kinase profiling data for each of these drugs.

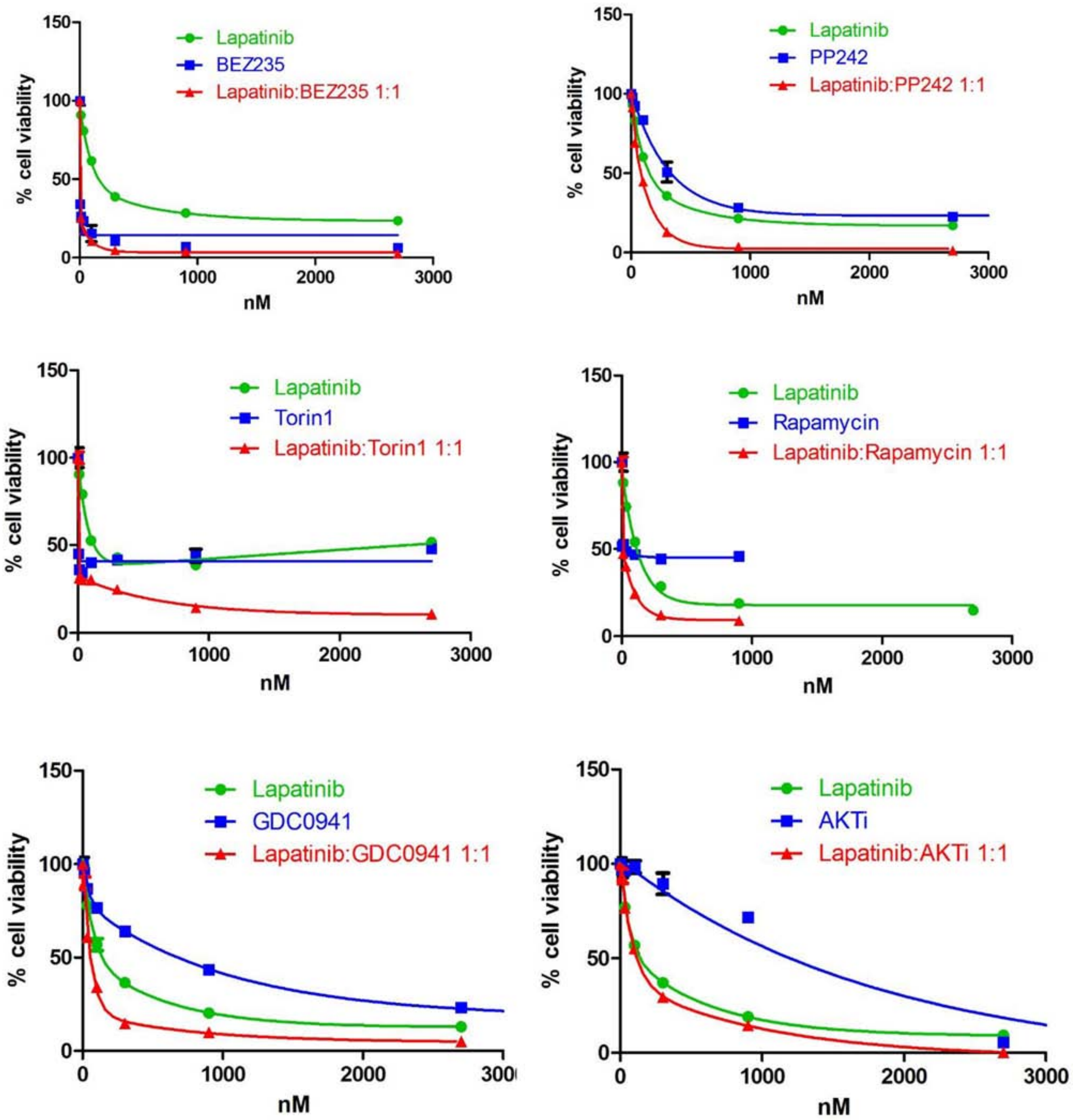

Supplementary Figure S2: Growth assays were done in matrix format. SkBr3 cells (2000/well) were plated in 96 well plates and were allowed to adhere overnight. The cells were treated with increasing doses of the indicated in a 3-fold increasing matrix format. Cell viability was determined at 72 hours by MTT assay. The percentage absorbance upon treatment compared to DMSO treatment is reported as an average of triplicates.

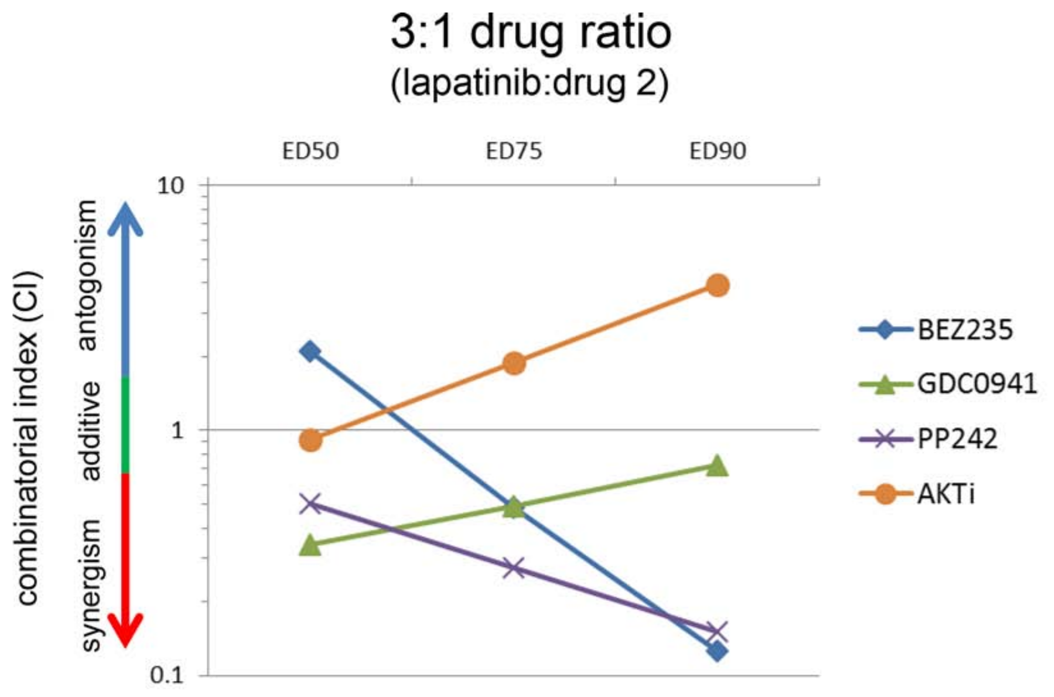

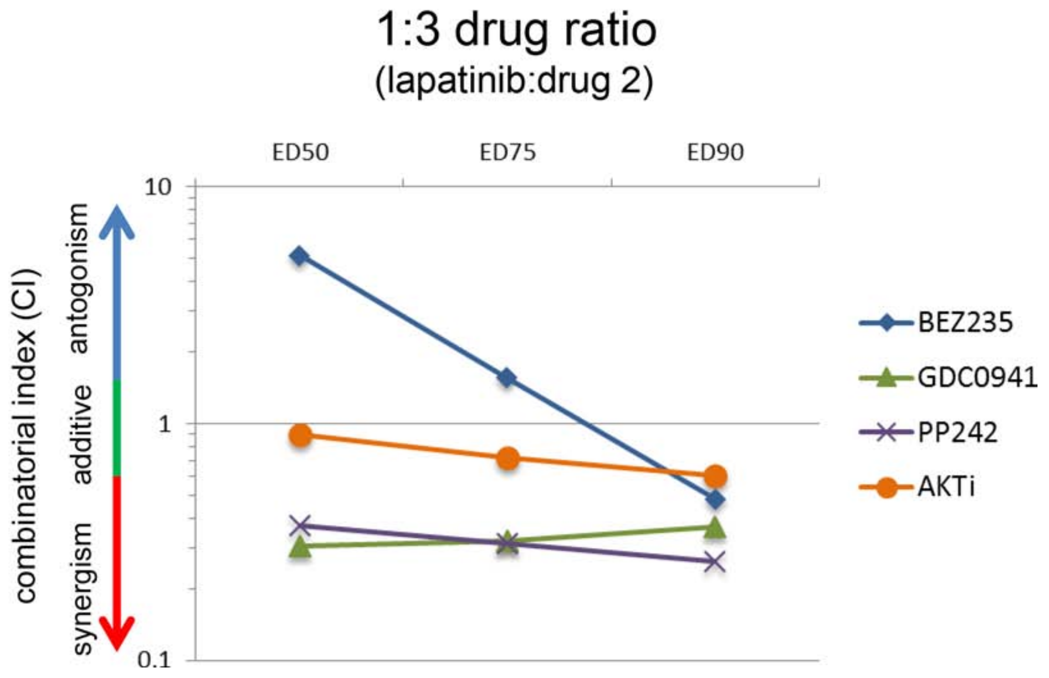

Supplementary Figure S3: Growth assays were done in matrix format. SkBr3 cells (2000/well) were plated in 96 well plates and were allowed to adhere overnight. The cells were treated with increasing doses of the indicated in a 3-fold increasing matrix format. Cell viability was determined at 72 hours by MTT assay. The percentage absorbance upon treatment compared to DMSO treatment is reported as an average of triplicates. The drugs were at fixed ratios of 1:3 or 3:1 for lapatinib:drugX. The Calcusynsoftware analyses for synergy, additivity or antagonism between two drug combinations based on the Chou & Talay methodology.

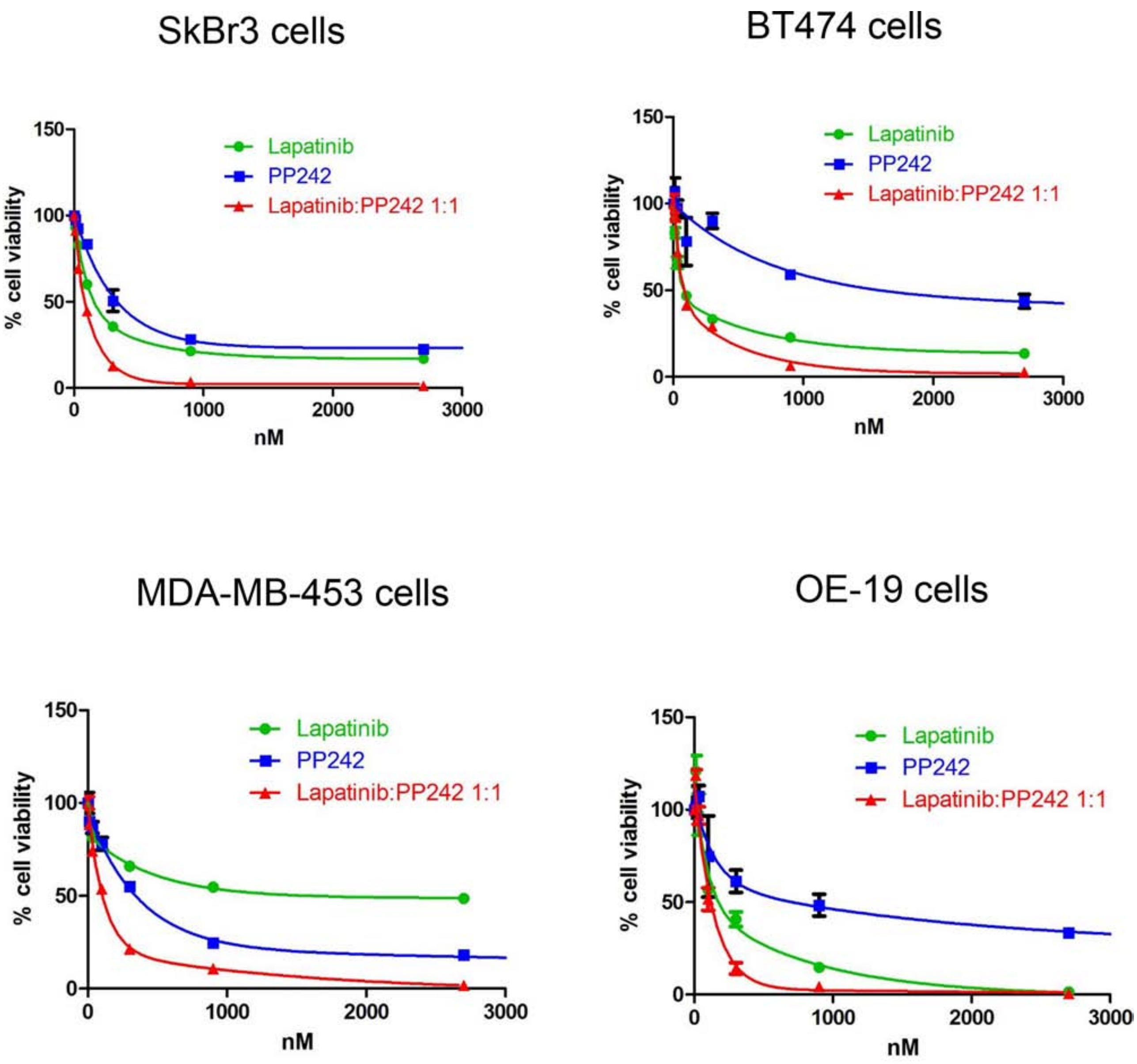

Supplementary Figure S4: Growth assays were done in matrix format for the indicated four cell types. Cells were treated with increasing doses of the indicated in a 3-fold increasing matrix format. Cell viability was determined at 72 hours by MTT assay. The percentage absorbance upon treatment compared to DMSO treatment is reported as an average of triplicates.

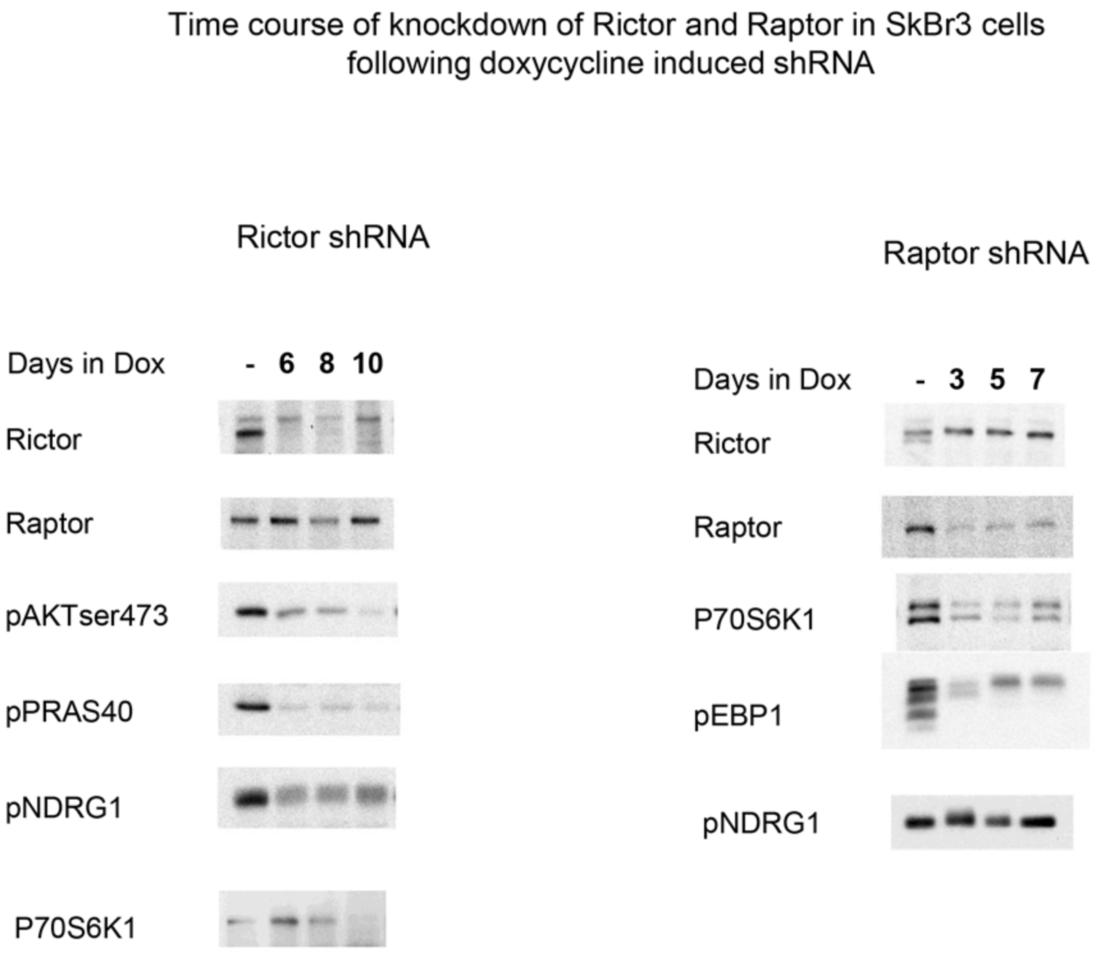

Supplementary Figure S5: SkBr3 cells engineered to induce either Rictor shRNA or Raptor shRNA were treated with 200 ng/ml doxycycline for the indicated number of days and cell lysates immunoblotted using the indicated antibodies.

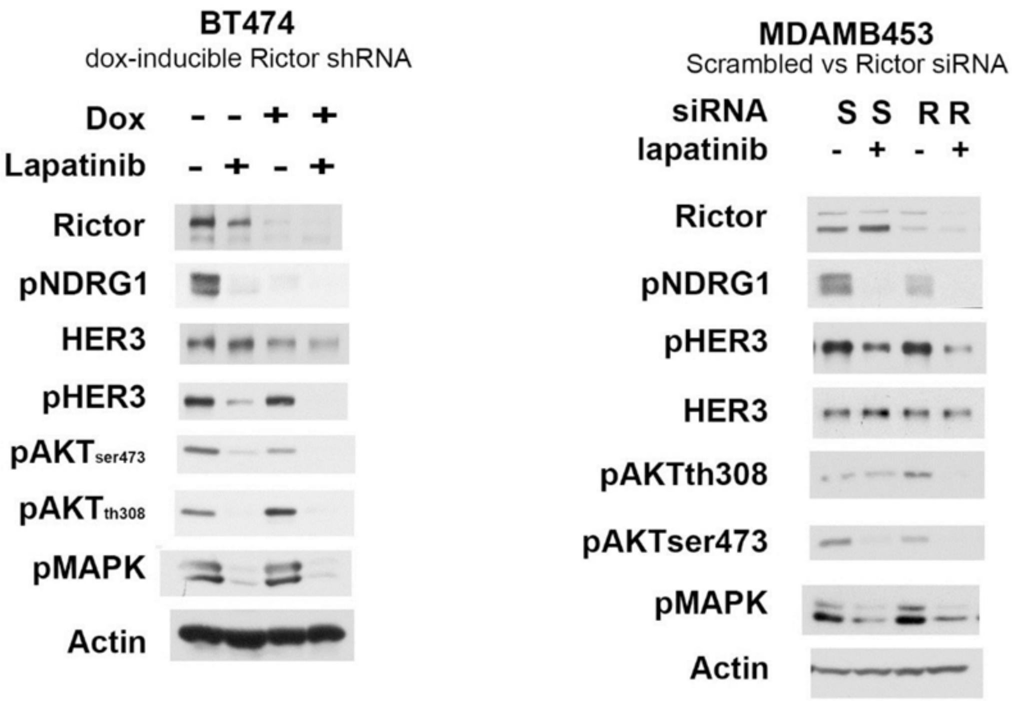

Supplementary Figure S6: Rictor knockdown inhibits the compensatory upregulationof HER3 and downstream signaling in BT474 and MDA-453 cells. BT474 cells were stably transfected with a dox-inducible RictorshRNA construct and treated with 200 nM lapatinibfor 72 hours following the dox-induced knockdown of Rictor. MDA-MB-453 cells were transiently transfected with Rictoror scrambled siRNA and treated with 200 nM lapatinibfor 72 hours.

Supplementary Table S1: IC50 values determined form curve fit of dose response for each drug

Drug	IC50 (nM)
lapatinib	243
GDC0941	601
BEZ235	42
AKTi	280
MK2206	>8100
PP242	495
Torin1	<3
Rapamycin	26
CI1040	1240
